# Inhibition of SDF-1 receptors CXCR4 and CXCR7 attenuates acute pulmonary inflammation via the adenosine A_2B_-receptor on blood cells

**DOI:** 10.1038/cddis.2016.482

**Published:** 2017-05-25

**Authors:** Franziska Magdalena Konrad, Nadine Meichssner, Annette Bury, Kristian-Christos Ngamsri, Jörg Reutershan

**Affiliations:** 1 Department of Anesthesiology and Intensive Care Medicine, University Hospital of Tübingen, Tübingen, Germany; 2 Department of Anesthesiology and Intensive Care Medicine, Hospital of Bayreuth, Germany

## Abstract

Acute pulmonary inflammation is characterized by migration of polymorphonuclear neutrophils into the different compartments of the lung. Recent studies showed evidence that the chemokine stromal cell-derived factor (SDF)-1 and its receptors CXCR4 and CXCR7 influence migration of immune cells and their activity was linked to adenosine concentrations. We investigated the particular role of CXCR4- and CXCR7-inhibition and the potential link to the adenosine A_2B_-receptor, which plays an important anti-inflammatory role in the lung. After LPS-inhalation for 45 minutes, administration of the CXCR4-inhibitor (AMD3100) decreased transendothelial and transepithelial migration, whereas CXCR7-antagonism influenced epithelial migration exclusively. In A_2B_−/− mice, no anti-inflammatory effects were detectible through either one of the agents. Using chimeric mice, we identified A_2B_ on hematopoietic cells to be crucial for these anti-inflammatory effects of CXCR4/7-inhibition. Both inhibitors decreased TNF*α*, IL6, CXCL1 and CXCL2/3 levels in the bronchoalveolar lavage of wild type mice, while not influencing the chemokine release in A_2B_−/− mice. Inflammation augmented the expression of both receptors and their inhibition increased A_2B_-levels upon inflammation. *In vitro* assays with human epithelium/endothelium confirmed our *in vivo* findings. During inflammation, inhibition of CXCR4- and CXCR7-receptors prevented microvascular permeability in wild type but not in A_2B_−/− mice, highlighting the pivotal role of an active A_2B_-receptor in this setting. The combination of both inhibitors had a synergistic effect in preventing capillary leakage. In conclusion, we determined the pivotal role of CXCR4- and CXCR7-inhibition in acute pulmonary inflammation, which depended on A_2B_-receptor signalling.

Acute pulmonary inflammation and its more severe form acute respiratory distress syndrome still have a high mortality around 40%^1^ and the surviving patients commonly have residual physical limitations and a poor quality of life.^2^

The innate inflammatory response to pathogens includes the release of chemotactic factors to recruit polymorphonuclear neutrophils (PMNs). Although PMNs are necessary for defense, their excessive migration into inflamed tissue even aggravates tissue damage.^3^ Thereby, PMNs migrate from the circulation into the lung interstitium passing an endothelial barrier followed by an epithelial barrier into the alveolar space.

Stromal cell-derived factor (SDF)-1 is a chemokine that mediates hematopoietic stem cell mobilization and migration of leukocytes.^4, 5^ SDF-1 is naturally highly expressed in the bone marrow and acts as a retention factor for neutrophils. During inflammation, the concentration of SDF-1 in the bone marrow decreases and PMNs enter the circulation from where they can migrate to the site of inflammation.^6^

SDF-1 (CXCL12 in the systematic nomenclature) has two receptors: CXCR4 and CXCR7.^7^ These receptors seem to play a role in lung emphysema and chronic obstructive pulmonary disease.^8^

The nucleoside adenosine emerges from the enzymatic degradation of adenosine triphosphate. Four different adenosine receptors exist, whereby the A_2B_-receptor plays a predominant role in terms of pulmonary inflammation.^9, 10^ A connection between the A_2B_-receptor and CXCR4-expression was also found in terms of protection against vascular injury.^11^

Therefore, we investigated the role of the SDF-1 receptors CXCR4 and CXCR7 concerning the two hallmarks of acute pulmonary inflammation: PMN migration and microvascular permeability. Additionally, we hypothesized that inhibiting CXCR4 and CXCR7 has anti-inflammatory effects and that these effects depend on A_2B_-receptor signalling.

## Results

### SDF-1 levels in our model

**Figure 1:**
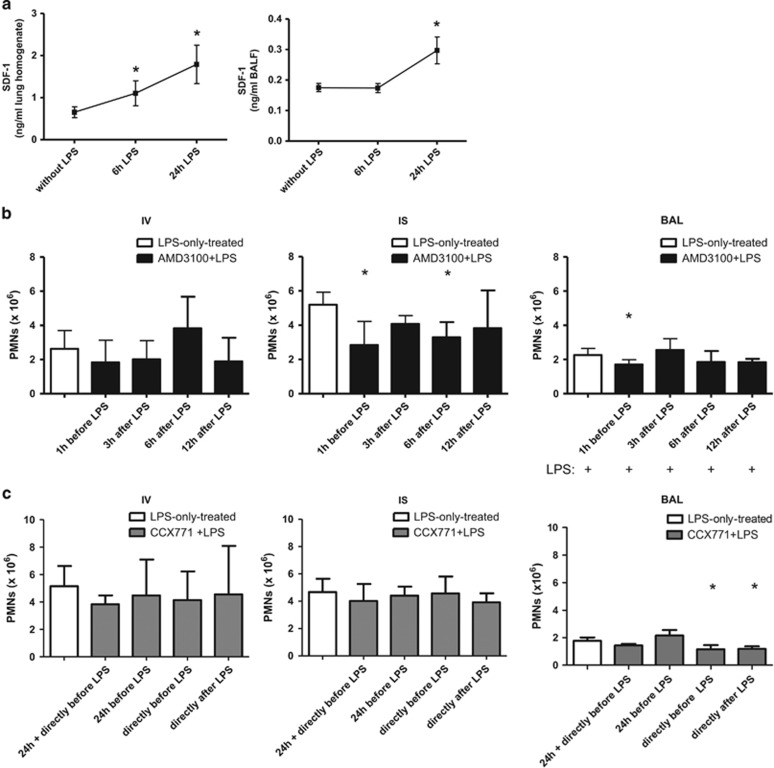
Effect of our model on SDF-1 levels in the lungs of mice (**a**). Mice inhaled LPS and SDF-1 levels were determined in the lungs (*n*=8) and BAL (*n*=4). Data are presented as mean ±S.D.; *P<0.05 *versus* without LPS. Time optimum for the administration of the CXCR4- (**b**) and CXCR7-antagonist (**c**). The inhibitors were given at indicated time points and, 24 h after LPS-inhalation, migration of PMNs into the different compartments of the lung (IV=intravascular; IS=interstitial; BAL=bronchoalveolar lavage) was evaluated. Data are presented as mean ±S.D.; *n*≥4; *P<0.05 versus LPS-only treated

We determined the impact of our model on SDF-1 levels in the lungs of mice and bronchoalveolar lavage (BAL) (Figure 1a). LPS-inhalation significantly increased SDF-1 in the lungs of mice 6 and 24 h after LPS. In the BAL, the significant rise of the chemokine was detectable 24 h after the inflammatory hit.

### Time-dependent administration of the CXCR4- and CXCR7-antagonist

Based on previously published data, AMD3100 was administered to the animals at different time points^12, 13^ and all animals inhaled LPS (Figure 1b). There were no differences in PMN counts in the intravascular compartment. In the interstitium of the lung, CXCR4-antagonism was effective in curbing down PMN migration 1 h before LPS and 6 h after the inflammation. One hour before LPS, AMD3100 decreased PMN migration into the alveolar space. Hence, we chose 1 h before LPS as time point for the administration of AMD3100 in all subsequent experiments.

We chose different time points for the administration of CCX771.^13, 14^ CXCR7-antagonism neither influenced PMN counts intravascular nor interstitial, but reduced migrated PMNs into the alveolar space when injected shortly before or directly after LPS (Figure 1c). Therefore, CCX771 was administered directly after LPS in all following experiments.

### The effect of CXCR4- and CXCR7-antagonism in immunohistochemistry

**Figure 2:**
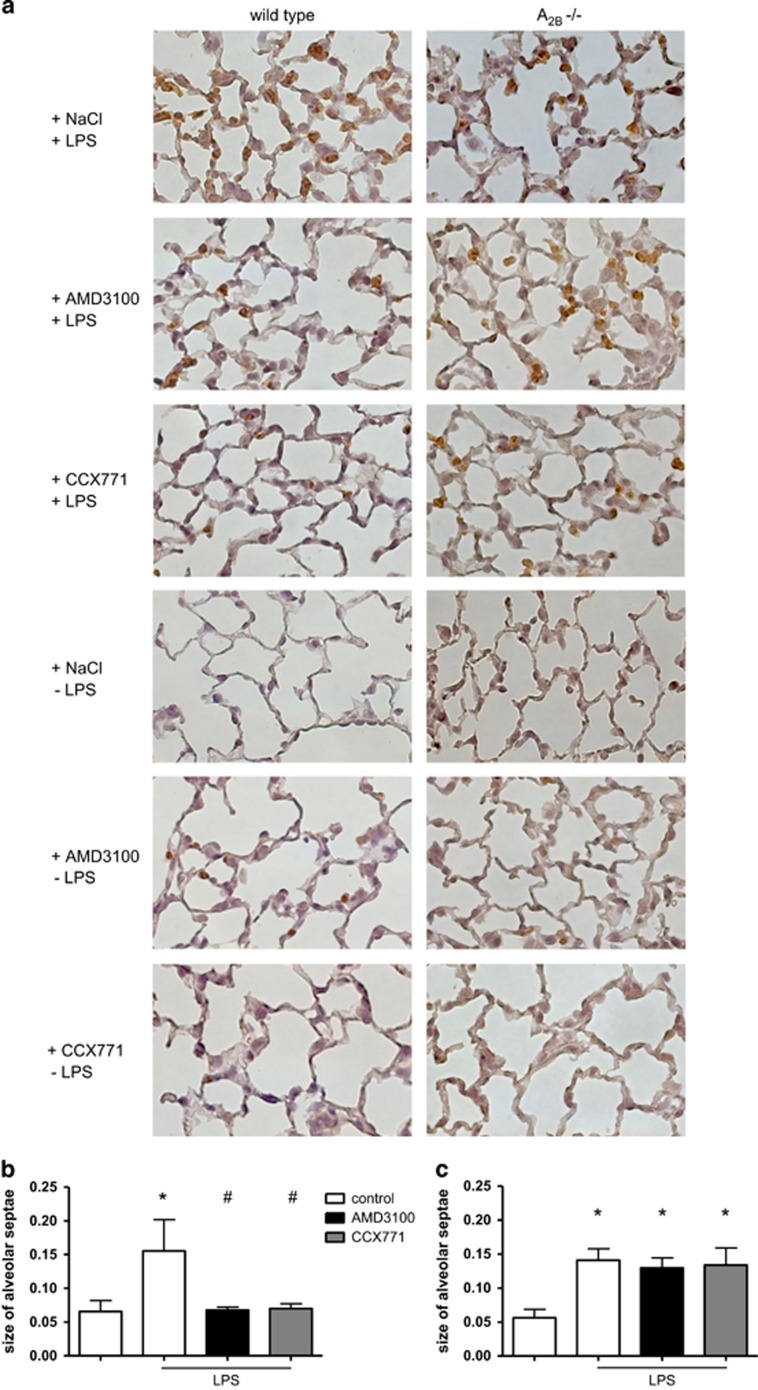
Impact of AMD3100 and CCX771 on PMN infiltration into the lungs and alveolar thickness identified by immunohistochemistry. Neutrophils were stained with a specific marker and appear brown in histology (rat anti-mouse neutrophil, clone 7/4) (original magnification, × 63). AMD3100 is the specific inhibitor of CXCXR4; CCX771 inhibits CXCR7. All conditions were investigated in wild type (left column) and A_2B_−/− animals (right column) (**a**). Images are representatives of *n*=4 experiments. Alveolar septae of the different conditions were measured in wild type (**b**) and A_2B_−/− animals (**c**). Data are presented as mean ±S.D.; *n*≥8; *P<0.05 versus control; ^#^*P*<0.05 versus LPS-treated

To visualize the effects of inhibiting the SDF-1 corresponding receptors on the architecture of the lung and quantitatively evaluate PMN migration, we prepared slides for immunohistochemistry where PMNs appear brown (Figure 2a). LPS-inhalation increased migrated PMNs and alveolar septae were significantly thickened (Figure 2b). AMD3100 and CCX771 both significantly decreased PMNs and the size of alveolar septae, confirming our results from the *in vivo* PMN migration assay. Without inflammation, both inhibitors did not affect the size of alveolar septae.

Following our hypothesis that an anti-inflammatory effect of CXCR4- and CXCR7-inhibition depends on A_2B_-receptor signalling, we performed immunohistochemistry slides also in A_2B_−/− mice. Corresponding to wild type animals, LPS-inhalation increased PMN migration into the lung interstitium and alveolar size rose (Figure 2c). Neither CXCR4- nor CXCR7-antagonism changed PMN counts and alveolar thickness in A_2B_−/− mice.

### *In vivo* PMN migration assay

To quantitatively determine the effect of AMD3100 and CCX771 on PMN migration, we performed an *in vivo* PMN migration assay and identified PMNs migrated into the different compartments of the lung by a flowcytometry-based method.

**Figure 3:**
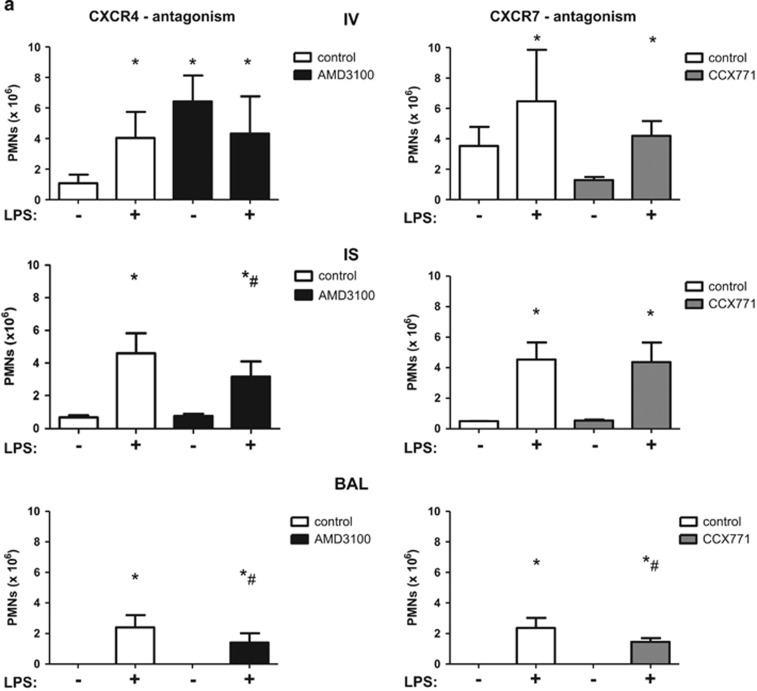

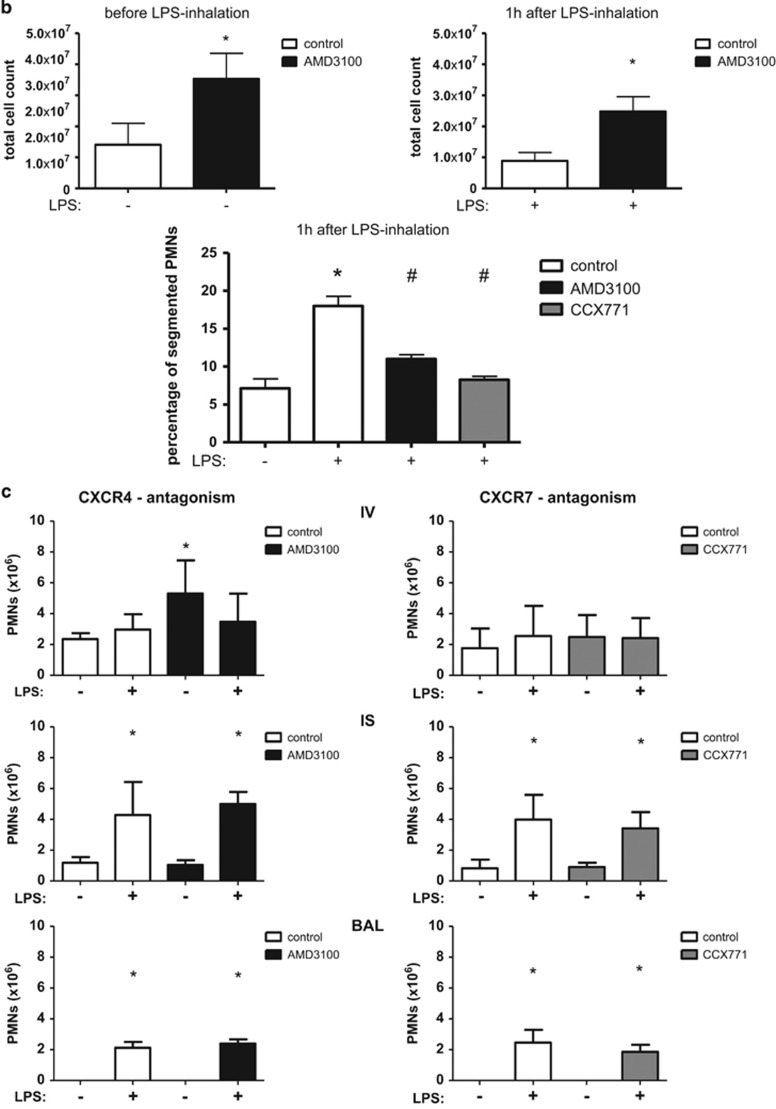
Effect of the inhibition of CXCR4 and CXCR7 on PMN migration into the lung. In wild type animals (**a**) and A_2B_−/− mice (**c**), AMD3100 (CXCR4-inhibitor) and CCX771 (CXCR7-inhibitor) were injected and migration of PMNs into the different compartments of the lung without and with LPS (IV = intravascular; IS = interstitial; BAL = alveolar space) quantified. Differential blood counts were performed at indicated time points and total cell counts and also segmented PMNs in wild type animals determined (**b**) (*n*≥3). Data are presented as mean ±S.D.; *n*=4 without LPS; *n*≥6 with LPS; *P<0.05 versus control; ^#^*P*<0.05 versus LPS-treated

In wild type animals, LPS-inhalation caused a rise of PMNs attached to the endothelium (Figure 3a). SDF-1 keeps PMNs in the bone marrow via CXCR4 and the antagonism of CXCR4 causes a release of neutrophils from the bone marrow in the circulation.^15^ Therefore, in our model, CXCR4-inhibition increased intravascular PMN counts significantly even without LPS-inhalation. The inhibition of CXCR7 did not lead to any changes in the intravascular compartment. In the interstitium of the lung, LPS caused a rise of PMN influx, whereas AMD3100 decreased migrated PMNs significantly. CCX771 did not influence interstitial PMN counts. In the alveolar space, LPS-inhalation increased migrated PMNs significantly. The reduction of PMN migration by the antagonism of CXCR4 was also apparent in the alveolar space and the inhibition of CXCR7 decreased alveolar PMN reflux.

To further investigate the fact that inhibition of CXCR4 increased intravascular PMN counts but, at the same time, reduced migrated PMNs into the lung interstitium and alveolar space, we performed differential blood counts. AMD3100 increased intravascular cell counts significantly even without inflammation, confirming our results from the *in vivo* migration assay (Figure 3b). LPS-inhalation increased the percentage of segmented and therefore mature PMNs intravascular. These PMNs are known to sequester preferentially into the lung capillaries.^6, 16, 17, 18^ Differential blood counts further revealed that the percentage of segmented and therefore mature PMNs was significantly decreased in CXCR4- and CXCR7-inhibited animals, explaining the reduced migration of PMNs into the alveolar space and confirming our results from the *in vivo* transmigration assay.

In A_2B_−/− mice, CXCR4-antagonism increased PMNs attached to the endothelium without LPS-inhalation (Figure 3c). In the interstitium of the lung, LPS enhanced PMN migration significantly, whereas neither CXCR4- nor CXCR7-inhibition changed these PMN counts. LPS caused a rise of the PMN influx into the alveolar space and, in A_2B_−/− mice, AMD3100 and CCX771 did not change transepithelial migration, indicating the pivotal role of the A_2B_−/− receptor in this setting and confirming our findings from immunohistochemistry.

### Influence of CXCR4- and CXCR7-antagonism on the release of chemokines

**Figure 4:**
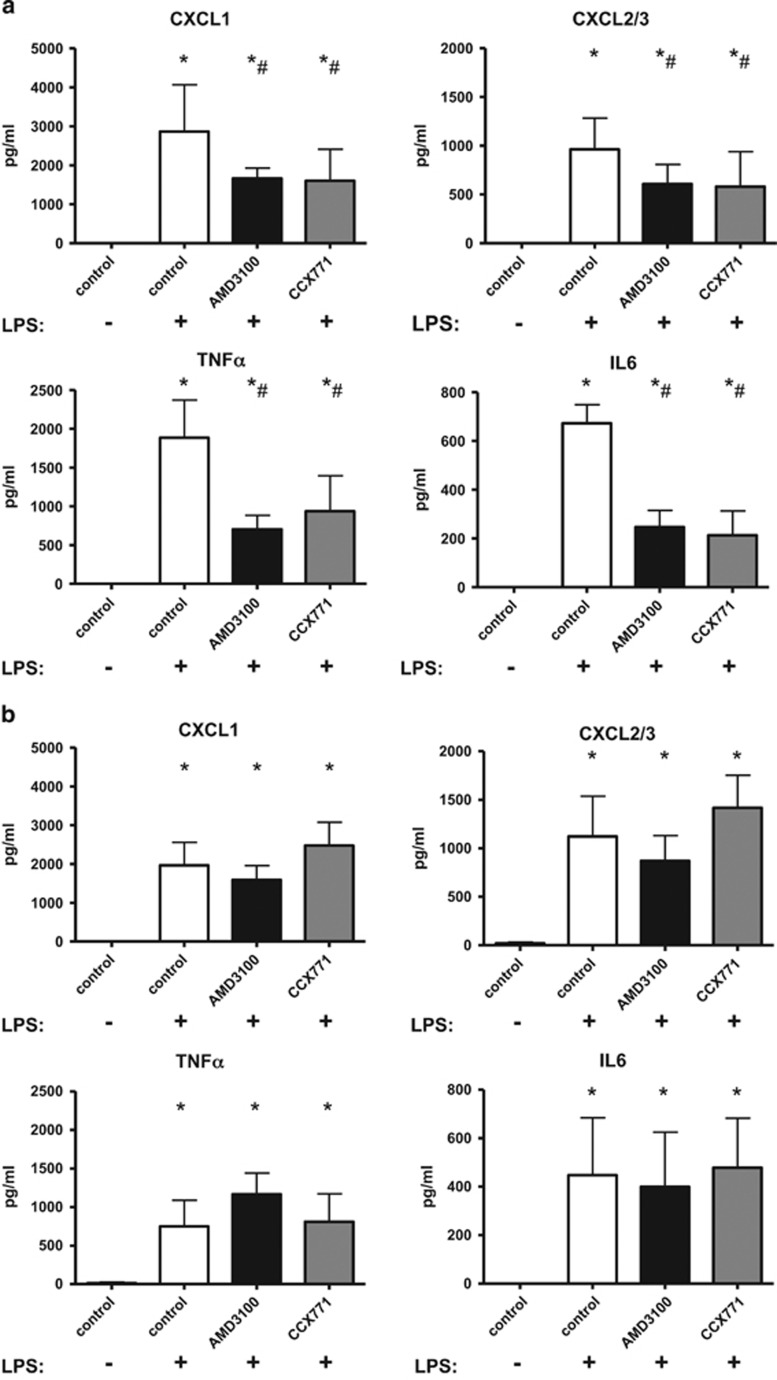
CXCR4- and CXCR7-antagonism and its effects on the release of chemokines. In wild type animals, LPS-inhalation increased all chemokine levels and the administration of both inhibitors decreased this effect (**a**). In A_2B_−/− animals, AMD3100 and CCX771 did not influence the release of chemokines (**b**). Data are presented as mean ±S.D.; *n*=4 without LPS; *n*≥7 with LPS; *P<0.05 versus control; ^#^*P*<0.05 versus LPS-treated

CXCL1 and CXCL2/3 are crucial for PMN migration into inflamed tissue, whereas TNF*α* and IL6 have a key role in orchestrating complex function and regulation of inflammation.^19, 20^ LPS caused a rise of all four chemokines in the BAL of mice (Figure 4). In wild type animals, inhibition of CXCR4 and, to a similar amount, CXCR7-antagonism decreased all four chemokines, confirming our results from the PMN migration assay (Figure 4a). In A_2B_−/− mice, LPS-inhalation significantly increased the chemotactic and inflammatory chemokines (Figure 4b). AMD3100 and CCX771 did not influence any chemokine in these mice, emphasizing again a critical role of the A_2B_−/− receptor on their mode of action.

### Influence of AMD3100 and CCX771 on different cell types

To differentiate between the anti-inflammatory effects of both inhibitors on different cell types, we performed an *in vitro* PMN transmigration assay. Thereby, freshly isolated human PMNs migrate through a monolayer of human pulmonary epithelial/endothelial cells and can be treated separately.

**Figure 5:**
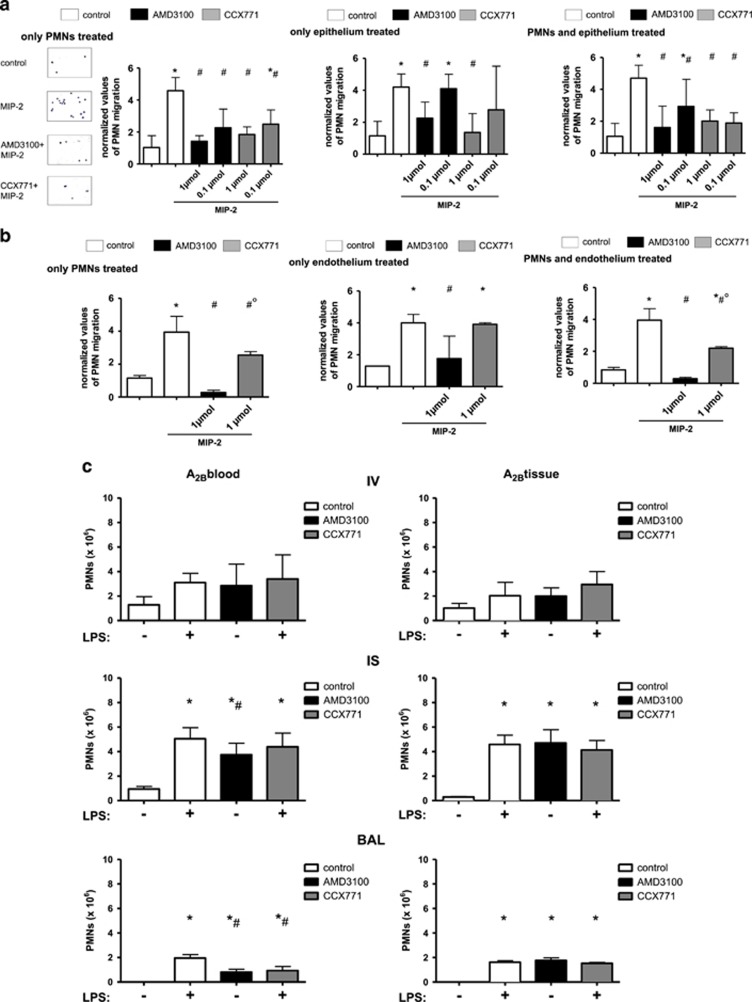

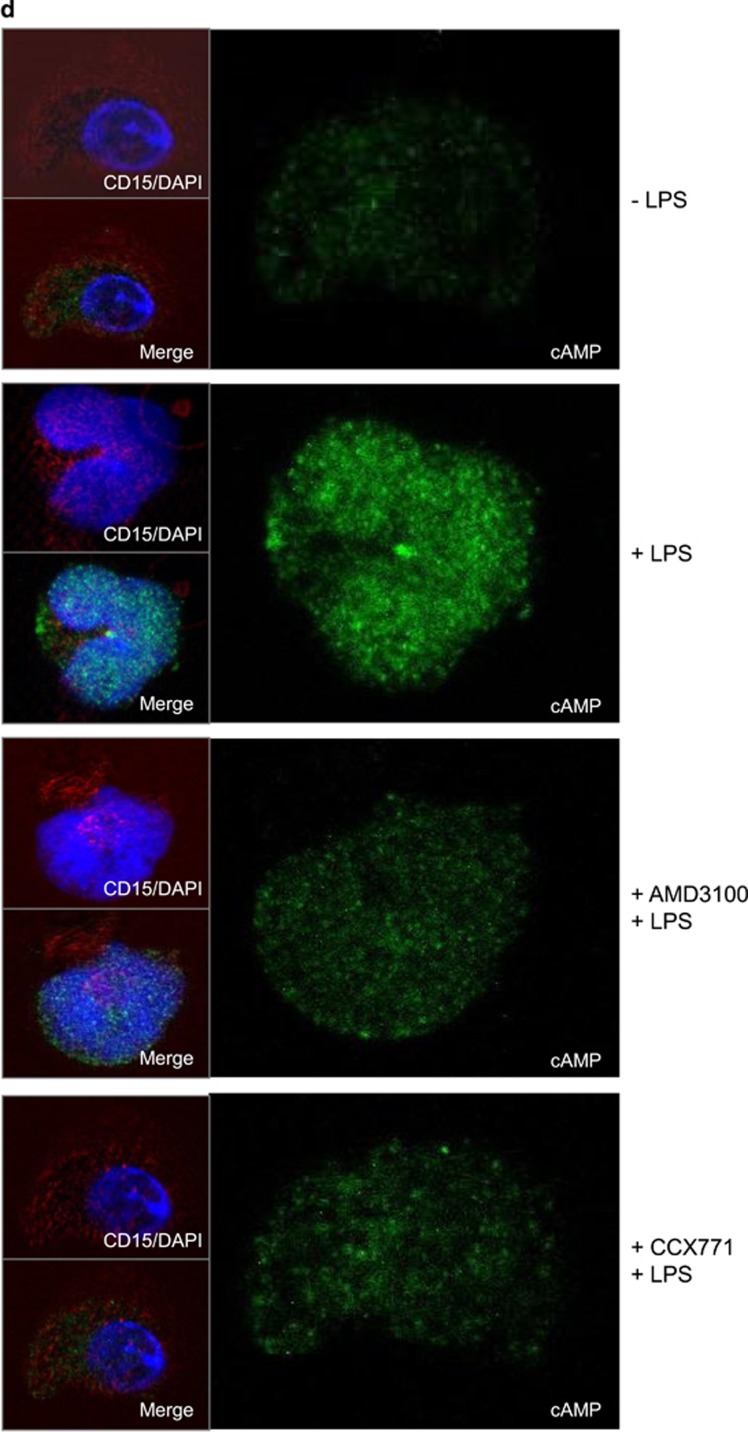
Influence of CXCR4 and CXCR7-inhibion on hematopoietic and non-hematopoietic cells. *In vitro* transmigration assay of human PMNs through a pulmonary epithelial (**a**) and endothelial (**b**) monolayer. PMNs, epithelium or endothelium were treated with CXCR4- (AMD3100) or CXCR7- (CCX771) inhibitors and migration of PMNs through a monolayer of human epithelium/endothelium measured. Migration of PMNs was initiated through the chemokine MIP-2 (CXCL2/3) at indicated wells. Representative pictures of modified Giemsa staining of migrated PMNs were shown to verify results from MPO-measurements (*n*≥3). Data are presented as mean ±S.D.; (**a**) *n*≥3; (**b**) *n*≥2; *P<0.05 versus control; ^#^*P*<0.05 versus MIP-2-treated; °*P*<0.05 versus AMD3100. In chimeric mice (**c**), migration of PMNs was determined in the different compartments of the lung (IV = intravascular; IS = interstitial; BAL = alveolar space). A_2B_−/− mice received bone marrow from wild type mice and possessed the A_2B_-receptor only on hematopoietic cells (A_2B_blood). Wild type mice received bone marrow from A_2B_−/− and expressed A_2B_ only on non-hematopoietic cells (A_2B_tissue). Data are presented as mean ±S.D.; *n*≥3 without LPS; *n*≥5 with LPS; *P<0.05 versus control; ^#^*P*<0.05 versus LPS-treated. Cyclic adenosine monophosphate (cAMP) in human PMNs after inflammation and CXCR4/7 treatment (**d**). Human PMNs were isolated and stimulated as indicated. Images are representatives of four experiments with similar results (original magnification, × 63). cAMP was stained with a specific antibody and appears green, nuclei were stained with DAPI and emerge blue

Isolated treatment of PMNs either with AMD3100 or CCX771 was effective in curbing down PMN migration through a pulmonary epithelial monolayer at two different concentrations (Figure 5a). Thereby, migrated PMNs were quantified by the determination of myeloperoxidase as described later. We added Giemsa-modified stained slides to the first experiment to prove statistical correctness. When only the epithelium was treated, the lower concentrations of both inhibitors were not effective anymore, indicating a distinct role of hematopoietic cells for CXCR4- and CXCR7-antagonism. Combined treatment of PMNs and epithelial cells showed a similar effect compared to PMN-only treatment.

Both inhibitors were effective in curbing down PMN migration when treated PMNs migrated through pulmonary endothelium (Figure 5b) and, under this condition, AMD3100 was significantly more effective than blocking CXCR7, confirming our *in vivo* PMN migration results. Corresponding to our results from the *in vivo* PMN migration assay, treatment with the CXCR7-inhibitor did not show any reduction in terms of PMN migration when only the endothelium was treated. Blocking CXCR4 reduced PMN counts after the endothelium was incubated. The combined treatment of endothelium and PMNs had no additive effect on PMN migration.

### The influence of hematopoietic and non-hematopoietic A_2B_-expression on CXCR4- and CXCR7-antagonism

To further evaluate CXCR4- and CXCR7-inhibition on different cell types, we evaluated their effects in terms of PMN migration in chimeric mice. Wild type mice received bone marrow from A_2B_−/− and therefore expressed A_2B_ only on non-hematopoietic cells (A_2B_tissue). A_2B_−/− mice received bone marrow from wild type mice and possessed the A_2B_-receptor only on hematopoietic cells (A_2B_blood). The *in vivo* PMN migration assay revealed that the inhibition of CXCR4 and CXCR7 only had anti-inflammatory effects when the A_2B_-receptor was expressed on hematopoietic cells (A_2B_blood) (Figure 5c). Confirming our previous findings from the *in vivo* migration assay, AMD3100 reduced interstitial and intra-alveolar PMN counts significantly, whereas CCX771 inhibited the PMN-influx into the alveolar system. The expression of the A_2B_-receptor on non-hematopoietic cells (A_2B_tissue) led to the same results as in A_2B_−/− with no anti-inflammatory effects of CXCR4- and CXCR7-inhibition.

### Effect of CXCR4/CXCR7-inhibition on the downstream signalling pathway of the A_2B_-receptor

Changes of cyclic adenosine monophosphate (cAMP) in human PMNs were determined after inflammation and CXCR4/CXCR7-antagonism, to further evaluate the impact of both receptors on the downstream signalling pathway of the A_2B_-receptor. Inflammation caused an increase of the cAMP level, which was attenuated by the inhibition of CXCR4 and CXCR7, confirming our results with chimeric mice (Figure 5d).

### Microvascular permeability

**Figure 6:**
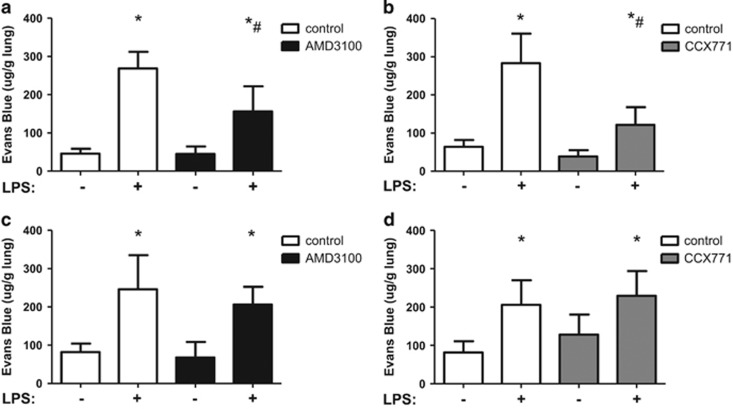
Microvascular permeability was attenuated by CXCR4- and CXCR7-inhibitors. Six hours after LPS-inhalation, the capillary leakage was assessed by Evans blue extravasation and the influence of CXCR4-antagonism (AMD3100) (**a**) (respectively CXCR7-antagonism (CCX771) (**b**)) in wild-type and A_2B_−/− animals (**c, d**) investigated. Data are presented as mean ±S.D.; *n*≥6; *P<0.05 versus control; ^#^*P*<0.05 versus LPS-treated

Evans blue extravasation was assessed as an indicator for capillary leakage, since microvascular permeability is the second hallmark of acute pulmonary inflammation besides PMN migration. LPS-treated mice showed a significant increase in capillary leakage (Figure 6). Administration of the specific CXCR4 (Figure 6a) or CXCR7 (Figure 6b) antagonist significantly decreased microvascular permeability, emphasizing their anti-inflammatory potential in stabilizing pulmonary barrier function.

In A_2B_−/− mice, LPS-inhalation increased capillary leakage (Figures 6c and d). AMD3100 (Figure 6c) and CCX771 (Figure 6d) failed to influence microvascular permeability, pointing out again the importance of an A_2B_−/− receptor signalling for the anti-inflammatory effects of CXCR4- and CXCR7-antagonism.

### Combined CXCR4- and CXCR7-inhibition

**Figure 7:**
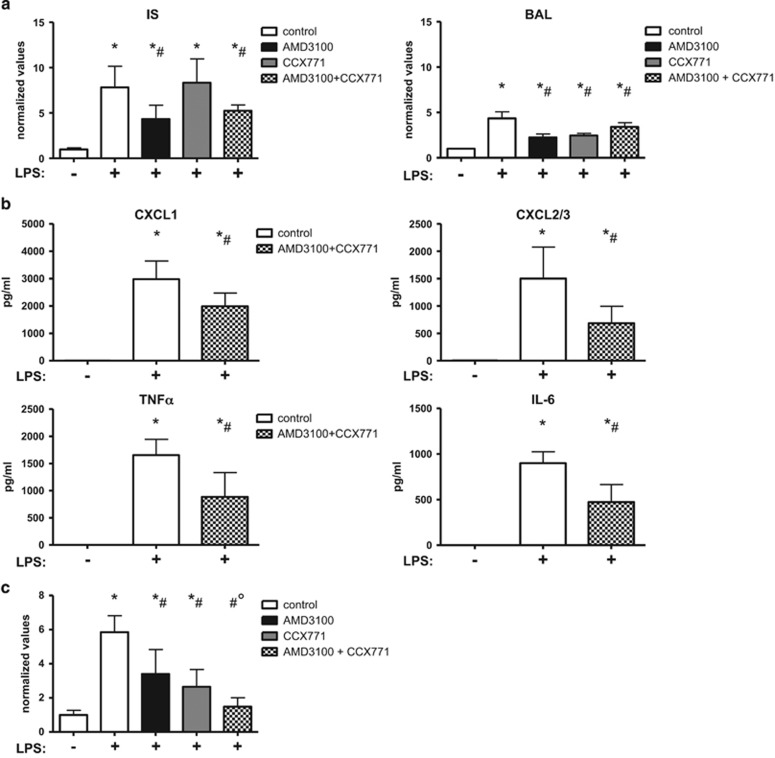
Combined inhibition of CXCR4 and CXCR7. The effect of simultaneous CXCR4- and CXCR7-antagonism was investigated in terms of PMN migration (IV=intravascular; IS=interstitial; BAL=bronchoalveolar lavage) (**a**), chemokine release (**b**) and microvascular permeability (**c**). There was no additive anti-inflammatory effect concerning PMN migration into the lung, but in terms of stabilizing the capillary leakage. Data are presented as mean ±S.D.; *n*≥6; *P<0.05 versus control; ^#^*P*<0.05 versus LPS-treated; °*P*<0.05 versus single treatment of AMD3100 or CCX771

It has been discussed whether CXCR4 and CXCR7 form a complex and may act additive.^14^ Therefore, we performed the PMN migration assay and combined the administration of AMD3100 and CCX771 (Figure 7a). The combined treatment showed no synergistic effect and was as effective as AMD3100 alone.

Confirming our results from the *in vivo* PMN migration assay and our results from the chemokine assay of the single treatments, additive CXCR4- and CXCR7-inhibition decreased all four chemokines significantly (Figure 7b).

Further on, we investigated the combined treatment in terms of microvascular permeability (Figure 7c). In this setting, simultaneous inhibition of CXCR4 and CXCR7 was significantly more effective compared to the inhibition of each receptor alone and almost completely prevented an increase of capillary leakage after LPS-inhalation.

### CXCR4/CXCR7- and A_2B_-gene expression and protein level in the lung

LPS-inhalation increased the expression of CXCR4 significantly (Figure 8.1). The inhibition of CXCR4 did not result in reduced expression of the receptor, whereas CXCR7-antagonism reduced CXCR4 levels as well as the synergistic administration of both inhibitors. These findings were confirmed on protein levels (Figure 8.2).

The expression of CXCR7 was also augmented during inflammation (Figure 8.1). AMD3100 increased CXCR7-expression even further, while CCX771 showed no effect. On protein level, both inhibitors did not lead to any change (Figure 8.3). The synergistic administration of both inhibitors reduced CXCR7-expression. The expression of both receptors was not influenced in A_2B_−/− mice—neither by inflammation nor by the administration of the specific inhibitors.

The expression of A_2B_ was increased after LPS-inhalation and by the administration of both inhibitors even further, highlighting again the link of CXCR4/CXCR7 and the A_2B_-receptor.

## Discussion

In humans, it has been shown that SDF-1 is expressed in the lung during acute lung injury.^13^ For the first time, we determined the pivotal role of the receptors of SDF-1—CXCR4 and CXCR7—in acute pulmonary inflammation and linked their anti-inflammatory potential to A_2B_-receptor signalling.

In the present study, we investigated a previously unknown anti-inflammatory effect of CXCR7 in acute pulmonary inflammation, predominantly in reducing transepithelial PMN migration into the alveolar space. Additionally, we are the first to determine the impact of CXCR4-inhibition on PMN migration into the different compartments of the lung. According to the findings of Petty *et al*,^13^ CXCR4-antagonism increased circulating PMNs, but decreased migrated PMNs into the interstitium of the lung and also the BAL, highlighting its anti-inflammatory potential. Thereby, inhibition of CXCR4 was also effective in decreasing interstitial PMN migration when given even 6 h after the inflammatory stimulus, verifying the role of SDF-1 in the later phase of PMN recruitment^13^ and also highlighting its clinical potential.

In the present study, the inhibition of CXCR4 and CXCR7 also showed a pivotal role in stabilization of the endothelial barrier. Both antagonists decreased the size of alveolar septae and minimized capillary leakage—the second hallmark of acute pulmonary inflammation.

Hypoxia induced the SDF-1/CXCR4 axis in multipotent stromal cells and increased their migration.^21, 22^ Additionally, hypoxia increased protein expression of CXCR4 and CXCR7 in the lung.^23, 24^ In our study on acute pulmonary inflammation, the expression of CXCR4 and CXCR7 also augmented as a reaction on hyper-inflammation. This is in accordance with the hypoxia-induced rise since inflammation is associated with tissue hypoxia, causing the so-called inflammatory hypoxia.^25^

In the present study, the results emphasized a previously unknown detrimental role of the A_2B_-receptor on SDF-1 signalling in acute pulmonary inflammation. Our findings indicate that the anti-inflammatory effects of CXCR4- and CXCR7-antagonism in terms of PMN migration, chemokine release and microvascular permeability are linked to adenosine A_2B_-receptor signalling. Inflammation increased the expression of CXCR4 and CXCR7 in wild type animals. A_2B_−/− mice express both receptors in the lung, but their expression was not influenced by inflammation or the inhibition of one of the receptors. Pointing to the same direction, the anti-inflammatory increase of the A_2B_-receptor as a reaction on inflammation^10^ was significantly augmented by inhibiting CXCR4 or CXCR7. Previous studies implicated a connection between adenosine and the chemokine SDF-1. It has been shown that extracellular adenosine triphosphate potentiated the chemotactic response to bone marrow-derived human mesenchymal stem cells and increased their migration.^26^ Dibutyryl cAMP increased expression of SDF-1 in wound tissue and enhanced endothelial progenitor cell migration.^27^ A link between CXCR4 and the A_2B_-receptor has been shown by a study about human colorectal carcinoma cells.^5^ Adenosine, which is present in the extracellular fluid of tumours because of their hypoxia, acted through A_2A_- and A_2B_-receptors to upregulate CXCR4-expression on tumour cells. A higher CXCR4-expression enables tumour cells to migrate towards SDF-1 and enhances proliferation and tumour dissemination.

The A_2B_-receptor plays a detrimental role in acute lung injury. Several studies showed that deletion of the receptor deteriorates the inflammation, whereas the administration of a specific A_2B_-receptor-agonist ameliorates tissue inflammation.^10, 28, 29, 30, 31^ In a previous study of our group, we identified the A_2B_-receptor on bone marrow cells as crucial for the anti-inflammatory effects of this receptor in our model of acute pulmonary inflammation.^10^ This is in accordance with the findings of the present study, where the anti-inflammatory effects of CXCR4- and CXCR7-antagonism depend on the expression of A_2B_ on blood cells. The findings of Yang *et al* also point to this direction, where chimeric mice with the expression of A_2B_ on blood cells showed less vascular lesion formation most probably due to reduced CXCR4 signalling.^11^

Sepsis changes adenosine affinity to its receptors and can further influence the receptor expression.^10, 32^ According to our results, patients with acute pulmonary inflammation, who would potentially benefit from inhibiting CXCR4 or CXCR7, should first be examined on their adenosine levels and receptor distribution. Considering our findings from the *in vivo* migration assay with chimeric mice, the expression of the A_2B_-receptor on hematopoietic cells is crucial. With the link of the anti-inflammatory effects of CXCR4/7-antagonism and A_2B_, we follow the recommendations from Dushiantan *et al*, who suggests to identify subgroups of patients where a specific treatment of pulmonary inflammation would be successful.^33^

## Conclusion

The presented data show a pivotal anti-inflammatory effect of inhibiting CXCR4 and CXCR7 in terms of PMN migration, chemokine release and microvascular permeability in acute pulmonary inflammation. We linked these anti-inflammatory effects to hematopoietic A_2B_-receptor signalling.

## Materials and methods

### Animals

C57BL/6 male mice were obtained from Charles River Laboratories (Germany) as corresponding wild type animals for A_2B_ gene-deficient mice (A_2B_−/−), which we received from Dr Katya Ravid (Boston University, School of Medicine, Department of Biochemistry, Boston, MA, USA). Mice were between 8 and 12 weeks old. All animal protocols were approved by the Animal Care and Use Committee of the University of Tübingen.

### CXCR4- and CXCR7-inhibitors

Ideal concentration of the specific CXCR4-inhibitor AMD3100 (Sigma-Aldrich, Germany) and CCX771, the specific CXCR7-inhibitor, (ChemoCentryx, CA, USA) were tested intraperitoneally respectively subcutaneoulsy based on the literature and company recommendations, respectively^12, 24^ (data not shown). The effect of the time-dependency of the inhibition of both receptors was evaluated (*n*≥4). Control mice received the solvent and close analogue of the antibody (ChemoCentryx).

### Murine model of acute lung injury

As described in detail before, 4–8 animals inhaled nebulized LPS from *Salmonella enteritidis* (Sigma-Aldrich) (a total of 7 ml, 500 μg/ml), which led to a reproducible acute pulmonary inflammation.^34, 35^

### Immunohistochemistry

Lungs were prepared as described previously (*n*=4).^10^ Since there were no differences between the solvent of AMD3100 and the vehicle control of CCX771 (data not shown), only one control is displayed in Figure 2.

### *In vivo* migration assay

Twenty-four hours after LPS-inhalation, we determined PMN migration into the different compartments of the lung via a flowcytometry-based method as described in detail before, including the gating process (*n*≥7).^36^ Briefly, fluorescent GR-1 (clone RB6-8C5) was injected into the tail vein of mice to mark all intravascular PMNs. To remove non-adherent leukocytes from the pulmonary vasculature, lungs were flushed free of blood by injecting saline into the beating right ventricle. PMNs from the alveolar space were obtained by BAL, which was performed with 5 ml NaCl. Lungs were homogenized and incubated with enzymes (hyaluronidase, collagenase and DNAse) for 30 min at 37 °C. Absolute cell counts were determined in the BAL and lungs. Fluorescent antibodies to CD45 (clone 30-F11) and 7/4 (clone 7/4) were added to the received cell suspension. All leukocytes were gated by their typical appearance in the forward/sideward scatter and further specified by their CD45 positive appearance. In the lung, we identified out of this CD45+-population intravascular PMNs, which were 7/4 positive and GR-1 positive. Interstitial PMNs were assigned as CD45 positive, 7/4 positive and GR-1 negative cells. In the BAL, CD45+ cells were further classified as 7/4 positive and GR-1 positive and identified as PMNs.

### Generation of chimeric mice

To identify whether the expression of the A_2B_-receptor on hematopoietic or non-hematopoietic cells is necessary for the anti-inflammatory effects of CXCR4- and CXCR7-inhibition, we generated chimeric mice (*n*≥5) as described previously.^10^ RT-PCRs of tissue and bone marrow were performed as control for successful transplantation (data not shown).

### Differential blood counts

Blood counts were performed from peripheral blood of the tail vein at indicated time points (modified Giemsa staining: Diff Quik; Dade Behring, Newark, DE, USA) (*n*≥3). Differential counts were conducted by two experienced independent observers by counting 100 leukocytes in randomly selected fields of view.

### Chemokine release

SDF-1 levels were determined in the supernatant of lung homogenates (*n*=8) and in the BAL (*n*=4) 6 and 24 h after LPS-inhalation according to the protocol of the manufacturer (Quantikine ELISA, R&D, Abingdon, UK).

Three hours after LPS-inhalation, the release of CXCL 1, CXCL 2/3, tumour necrosis factor-*α* (TNF*α*) and interleukin-6 (IL-6) were measured in the BAL of mice (*n*≥6).^37^

### Microvascular leakage

Evans blue extravasation was determined as a marker of capillary leakage.^37^ Evans blue (20 mg/kg, Sigma Aldrich, Steinheim, Germany) was injected into the tail vein 6 h after LPS exposure (*n*≥6).^10^ Thirty minutes later, intravascular Evans blue in the lungs was removed by flushing the beating right ventricle. Lungs were homogenized and Evans blue was extracted with formamide and the final concentration determined colorimetrically.

### CXCR4- and CXCR7 expression and protein level

We determined the expression of CXCR4, CXCR7 and A_2B_ in lungs of mice by RT-PCR (*n*≥7). The method was performed with the following primers CXCR4 (5′-CCC CGA TAG CCT GTG GAT-3′ and 5′-AGG ATG ACT GTC GTC TTG AGG G-3′), CXCR7 (5′-GGA GCC TGC AGC GCT CAC CG-3′ and 5′-CTT AGCCTG GAT ATT CAC CC-3′), and A_2B_ (5′-GCA TTA CAG ACC CCC ACC AA-3′ and 5′-TTT ATA CCT GAG CGG GAC GC-3′) as described.^37^

To further verify results from gene expression on protein level, we measured light intensity of fluorescent slides of lungs of mice. Rabbit polyclonal anti-CXCR4 and goat polyclonal anti-CXCR7 were used as primary antibody (Santa Cruz Biotechnology, Dallas, TX, USA). Images were visualized by using a confocal microscope (LSM 510, Meta, Carl Zeiss, Jena, Germany) and analysed by ZEN 5.0. Images are representatives of four experiments and were analysed using imageJ, a public programme being developed at the National Institutes of Health to officially analyse scientific images.

### *In vitro* PMN migration

We performed the *in vitro* transmigration assay of human PMNs through a monolayer of pulmonary epithelial (NCI-H441, ATCC, USA) (*n*≥3) or primary pulmonary endothelial cells (HMVEC-L, Lonza Walkersville, Walkersville, MD, USA). Endothelial (respectively epithelial) cells, PMNs, or both were incubated with the specific CXCR4 or CXCR7 antagonist at indicated concentrations. Human endothelial/epithelial cells were cultivated on inserts of a transwell system (3.0-μm pore size, 6.5-mm diameter; Costar, Cambridge, MA, USA) until reaching confluence. Isolated human PMNs (Percoll gradient; GE Healthcare Bio-Sciences AB, Uppsala, Sweden) migrated through the monolayer of endothelial/epithelial cells along a chemotactic gradient (CXCL2/3; 200 ng/ml; Pepro Tech, Hamburg, Germany). Migrated PMNs were quantified by determination of myeloperoxidase (absorption length: 405 nm) in the bottom wells. Additionally, migrated PMNs were evaluated by modified Giemsa staining (Diff Quik) by two experienced independent observers.

### Influence of CXCR4/CXCR7 inhibition on cAMP levels in human PMNs

Isolated human PMNs (Percoll gradient; GE Healthcare Bio-Sciences) were treated with the specific CXCR4/CXCR7 antagonist (1 μmol) and LPS (1 μg/μl). Anti-cAMP antibody (Abcam, Cambridge, UK) was used as primary antibody. Images were visualized by using a confocal microscope (LSM 510, Meta, Carl Zeiss) and analysed by ZEN 5.0. Images are representatives of four experiments and were analysed using imageJ, a public programme being developed at the National Institutes of Health to officially analyse scientific images.

### Statistical analysis

Data are presented as mean±S.D. unless indicated otherwise. Statistical analysis was performed using GraphPad Prism version 5.3 for Windows (GraphPad Software, San Diego, CA, USA). Differences between the groups were evaluated by one-way ANOVA followed by Bonferroni *post hoc* test. *P*<0.05 was considered statistically significant.

## Figures and Tables

**Figure 8:**
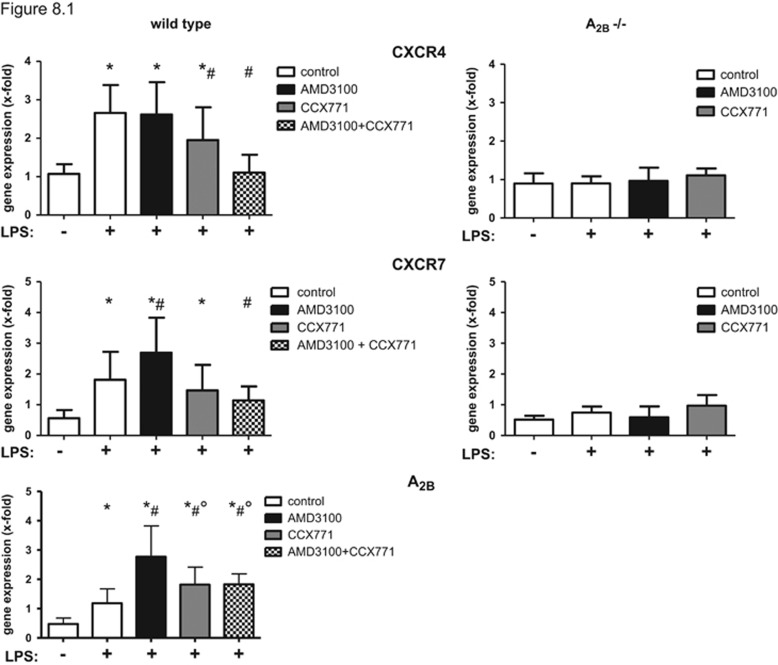

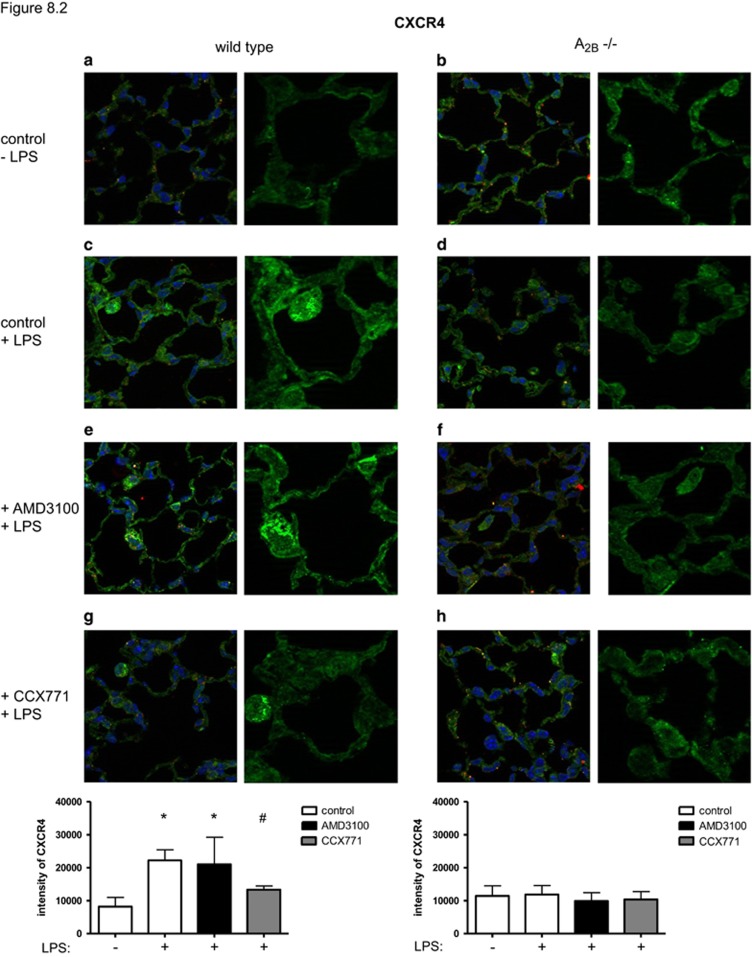

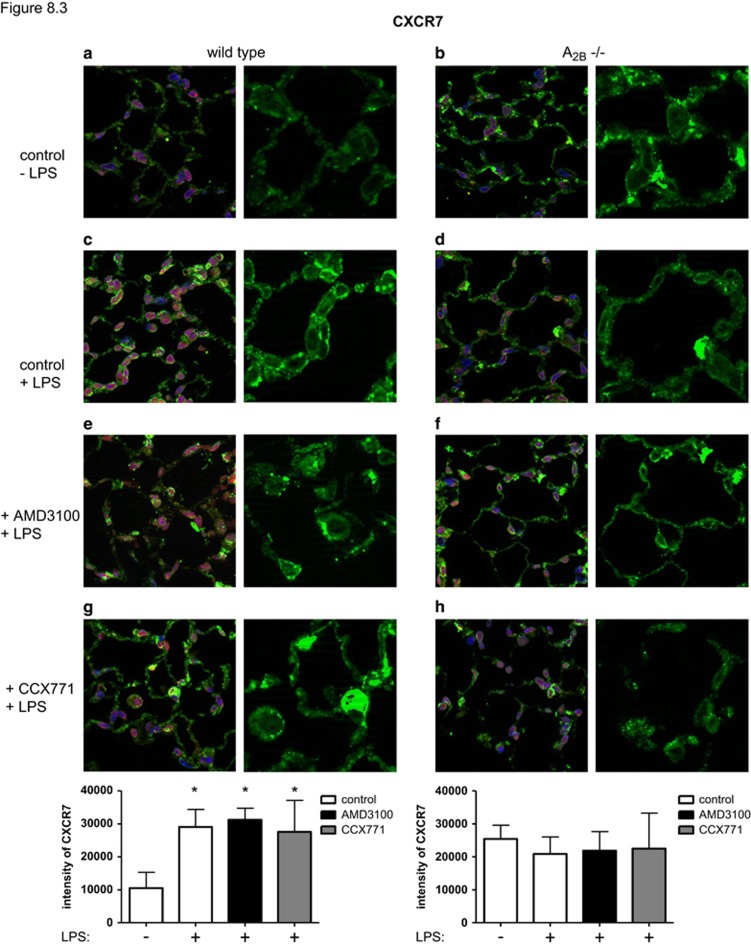
The impact of inflammation, CXCR4- and CXCR7-antagonism on the expression of both receptors and the A_2B_-receptor. LPS induced CXCR4 (*n*≥7), CXCR7- (*n*≥7) and A_2B_-gene expression (*n*≥6) in lungs of mice (8.1). Images are representatives of four experiments with similar results (original magnification, × 63). CXCR4 (8.2) (respectively CXCR7 (8.3)) were stained with specific antibodies and appear green, nuclei were stained with DAPI and emerge blue. Data are presented as mean±S.D.; *n*≥6; *P<0.05 versus control; ^#^*P*<0.05 versus LPS-treated; °*P*<0.05 versus single treatment of AMD3100
